# Successful and unsuccessful cannabis quitters: Comparing group characteristics and quitting strategies

**DOI:** 10.1186/1747-597X-6-30

**Published:** 2011-11-11

**Authors:** Sally E Rooke, Melissa M Norberg, Jan Copeland

**Affiliations:** 1 National Cannabis Prevention and Information Centre, University of New South Wales, 22-32 King Street, Randwick NSW 2031, Australia

## Abstract

**Background:**

In order to improve treatments for cannabis use disorder, a better understanding of factors associated with successful quitting is required.

**Method:**

This study examined differences between successful (*n *= 87) and unsuccessful (*n *= 78) cannabis quitters. Participants completed a questionnaire addressing demographic, mental health, and cannabis-related variables, as well as quitting strategies during their most recent quit attempt.

**Results:**

Eighteen strategies derived from cognitive behavioral therapy were entered into a principal components analysis. The analysis yielded four components, representing (1) Stimulus Removal, (2) Motivation Enhancement, (3) (lack of) Distraction, and (4) (lack of) Coping. Between groups comparisons showed that unsuccessful quitters scored significantly higher on Motivation Enhancement and (lack of) Coping. This may indicate that unsuccessful quitters focus on the desire to quit, but do not sufficiently plan strategies for coping. Unsuccessful quitters also had significantly more symptoms of depression and stress; less education; lower exposure to formal treatment; higher day-to-day exposure to other cannabis users; and higher cannabis dependence scores.

**Conclusions:**

The findings suggest that coping, environmental modification, and co-morbid mental health problems may be important factors to emphasize in treatments for cannabis use disorder.

## Background

Cannabis is the most commonly used illicit drug in the world, with a global estimate of 166 million users [1]. While some individuals are able to quit using cannabis when they want to, others experience greater difficulty, such that 9-15% of users become dependent upon cannabis [2,3]. Although a growing number of studies indicate that cognitive-behavioral and motivational interventions hold promise for treating cannabis use disorders [4-7], low abstinence and reduction rates signify that treatments need to be improved. In order to do so, a better understanding of factors associated with successful quitting is required. The current study, therefore, sought to examine the characteristics of successful and unsuccessful quitters, along with the strategies they used during their most recent quit attempt. This is the first study to make comparisons between frequent cannabis users who succeeded versus failed at a quit attempt within a community setting.

While a number of previous studies have examined predictors of cannabis use cessation, these studies either took a longitudinal perspective without restricting their examination to regular users or users with a desire to quit, focused solely on individuals within a treatment setting (who may differ largely from other regular cannabis users, most of whom do not seek professional help) [3], or looked at self-managed change without the inclusion of unsuccessful quitters as a comparison group. Longitudinal studies of individuals who have ever tried cannabis have found that continued use is associated with being male [8,9], younger age [8-10], being unmarried [8,10-12], lower educational achievement or unemployment [8,11], higher degree of use [9,10,13], a social context encouraging drug use [12,13], and depressive symptoms [9]. Studies of "self-managed change" or "natural" recovery that have not examined a comparison group have found that the most frequently reported quitting strategies are engaging in activities that are unrelated to cannabis use, making lifestyle changes, avoiding triggers for cannabis use, and seeking social support [14,15].

Studies focusing on cannabis users seeking or receiving professional treatment support the self-managed change literature [16,17], and add the additional findings that relapse is related to early lapses [18], a recovery environment in which drug use is not discouraged [16,17], and high levels of aversive psychological symptoms [19-22]. One randomized controlled trial examining outcome differences between combined motivational interviewing (MI) and cognitive behavioral therapy (CBT) and MI alone found that greater use of CBT-related methods predicted reductions in cannabis use [5]. Thus, the accumulating literature suggests that certain types of individuals (e.g., women, married people), along with individuals who develop and utilize adaptive quitting strategies may be more successful at abstaining from using cannabis.

### Research Objectives and Study Hypotheses

Identification of differences between successful and unsuccessful cannabis quitters potentially could improve interventions so that individuals more strongly oriented for relapse (or unable to quit for even short durations) might be better helped. In order to identify which individuals may be at greatest risk for relapse and what techniques may be most effective for achieving abstinence, an online survey was developed to compare the characteristics, as well as quitting strategies, of former regular cannabis users who had been abstinent for at least a year with current regular cannabis users who had made at least one unsuccessful attempt to quit. Research hypotheses were formulated based on the premise that the findings discussed above in relation to cannabis use cessation would extend to a sample of cannabis users who have not previously been studied in this context (i.e., frequent cannabis users who succeeded versus failed at a quit attempt within a community setting). The first hypothesis proposed that demographic variables commonly found to predict continued cannabis use would be associated with unsuccessful quitting in the current sample. These included younger age, being unmarried, having less education, and being male. Similarly, several cannabis-related variables were hypothesized to be associated with unsuccessful quitting, including having a higher degree of use and dependence, and having higher exposure to other cannabis users (Hypothesis 2). Based on the treatment study findings of Litt et al. [5], Hypothesis 3 predicted that successful quitters would report significantly higher use of cognitive-behavioral and motivational enhancement strategies than unsuccessful quitters. Finally, Hypothesis 4 predicted that unsuccessful quitters would report higher levels of depression, anxiety and stress than would successful quitters.

## Method

### Participants

Individuals eligible to participate in the study were aged 18 or older and either:

(a) previously used cannabis at least once a week for at least a year, but had not used any cannabis in the last year, or (b) currently used cannabis at least once a week and had made at least one unsuccessful attempt to quit. Eighty-eight successful and 82 unsuccessful Australian cannabis quitters were recruited through newspaper and online advertising (e.g., forums, Google advertising) from May 2009 to January 2010.

### Measures

Demographics. Demographic information included age, gender, ethnicity (Aboriginal/Torres Strait Islander, Anglo/Celtic, Asian, or other), marital status (defined as married/living with a partner versus single, dating, separated/divorced, or widowed [11]; and university education (yes or no). For individuals aged 21 or younger, we used the highest level of parental education, as has previously been done in studies involving participants not yet old enough to have attained their highest level of education [23]. An exception to this was when the participant's level of education exceeded that of his or her parents, in which case we used the participant's highest level of education.

Cannabis use. Information relating to participants' cannabis use included age of initiation, frequency of use (days per week) before most recent quit attempt, lifetime number of quit attempts, and past involvement in formal treatment for cannabis use (yes or no). Questions relating to exposure to other cannabis users, both at the time of survey completion and during the most recent quit attempt, included the following: "Do you currently live with another person who uses cannabis? During your most recent quit attempt, did you live with another person who used cannabis? Around how often do other people use cannabis in your presence? During your most recent quit attempt, how often did other people use cannabis in your presence? Around how often do people currently use cannabis in your house? During your most recent quit attempt, how often did other people use cannabis in your house?" Questions were rated on a five-point scale, where 1 = no/never, and remaining response options related to frequency of exposure (2 = 1-11 times a year, 3 = 1-3 days a month, 4 = 1-3 days a week, and 5 = 4-7 days a week). Responses to these six items were averaged to form a measure of exposure to other users. Participants whose average score was below 4 (i.e., less than weekly exposure) were categorized as having infrequent exposure, and those with an average score of 4 or higher (i.e., at least weekly exposure) were categorized as having frequent exposure.

Cannabis dependence. Participants retrospectively reported their level of cannabis dependence just prior to their most recent quit attempt using the five-item Severity of Dependence Scale (SDS) [24]. The measure has demonstrated good psychometric properties in previous research [25,26]. Cronbach's α was .90 in the current study.

Change strategies. Behavioral and experiential change processes during the participant's most recent quit attempt were assessed using the 20-item version of the Processes of Change questionnaire [27,28]. Originally developed to assess processes of change relating to tobacco smoking cessation, the measure was modified so that items referred to cannabis rather than tobacco [5]. The measure contained 10 items relating to behavioral change processes (e.g., "I read articles dealing with the problem of quitting cannabis"; "I used reminders to help me not to smoke"; " I had someone who listened when I needed to talk about my cannabis use") and 10 items relating to experiential change processes (e.g., "I felt more competent when I decided not to use cannabis"; "I considered the idea that people around me would be better off if I didn't use cannabis"; "Stories about cannabis and its effects upset me"). Response options were on a five point scale where 1 = never, 2 = seldom, 3 = occasionally, 4 = frequently, and 5 = repeatedly. The measure has predicted reductions in cannabis use in a previous study [5]. In the current study, Cronbach's α was .85 for behavioral processes and .84 for experiential processes. Because the Processes of Change measure does not cover many quitting strategies used in CBT-based treatments for cannabis, we also examined participants' use of 18 key quitting strategies derived from CBT/MI treatment programs for cannabis (e.g., strategies used in Copeland and colleagues' treatment package [4]). Response options were on the same five-point scale as the Processes of Change questionnaire. A principal components analysis of these items is reported in the Results section.

Psychological distress. Psychological distress at the time of survey completion was measured using the short-form of the Depression Anxiety Stress Scale (DASS-21) [29]. This 21-item measure contains three subscales assessing symptoms of depression, anxiety, and stress over the past week. Previous research supports the reliability and validity of this measure [30]. For the current study, Cronbach's α was .91, .86, and .90 for the depression, anxiety and stress scales, respectively.

### Procedure

The research received ethical approval from the University of New South Wales Human Research Ethics Committee (HREC; #09020). The sample was recruited through advertisements seeking individuals 18 years and older who either: (a) previously used cannabis at least once a week for at least a year, but had not used any cannabis in the last year, or (b) currently used cannabis at least once a week, and had made at least one unsuccessful attempt to quit. The advertisement provided a web address to the study information sheet, consent form, eligibility questions (age, quitting status), and survey. Forty-four individuals who did not fall into either of the required quitting status categories were automatically discontinued from the survey. Participants entered their postal addresses at the end of the survey, and were reimbursed for their time with a gift voucher worth $AUD20.

### Statistical Analyses

Five cases with missing data exceeding 20% were removed from the study. Four cases had missing data at levels between 6.7% and 12%. All other cases had less than 5% missing data. Individual variables had low levels of missing data (maximum = 5.3%). All missing values were imputed using SPSS 17's Expectation Maximization procedure, a maximum likelihood approach that employs an iterative algorithm estimating the parameters of the complete dataset [31].

The 18 CBT and MI quitting strategies were entered into a principal components analysis. The ratio of cases to variables was adequate for this analysis [32]. The first two research hypotheses were tested using binary logistic regression, while the second two hypotheses were tested using MANOVAs with follow-up univariate tests. All analyses contained several univariate and multivariate outliers. Results did not differ when outliers were removed from analyses; therefore, outliers were retained without adjustment.

## Results

### Descriptive Statistics and Correlations

The sample consisted of 99 males and 66 females. Ages ranged from 18-70 with a mean of 28.07 (*SD *= 10.17). The majority of participants were Anglo/Celtic (67.3%); 18.2% reported Asian ethnicity, and the remaining 13.9% reported being of another ethnicity. Table 1 shows group means and percentages on variables used in the analyses. Correlations among continuous variables in the analysis are shown in Additional File 1.

**Table 1:** Group Means and Percentage Scores on Outcome Variables

Measure	Quitter mean (SD) or %	Non-quitter mean (SD) or %	*df*	*t *	χ^2^	*p*
Age	28.38 (11.36)	27.60 (8.62)	164	0.50		.62
Age/initiation	16.91 (4.86)	15.77 (2.91)	164	1.81		.72
Use (days/wk)	3.79 (2.31)	4.81 (2.22)	164	2.87		<.01
Quit attempts	6.18 (15.86)	6.45 (12.07)	164	0.12		.09
SDS	2.26 (0.84)	2.60 (0.75)	164	2.67		<.01
POC Behavioral	2.83 (0.96)	2.63 (0.65)	164	1.55		.12
POC Experiential	2.69 (0.92)	2.62 (0.65)	164	0.50		.61
Depression	26.44 (11.07)	32.44 (11.64)	164	3.39		<.01
Anxiety	26.51 (10.86)	29.12 (9.31)	164	1.65		.10
Stress	29.85 (11.33)	34.33 (10.66)	164	2.60		.01
Gender (male)	57.5%	62.8%	1		0.49	.48
Married	29.9%	23.1%	1		0.98	.32
Uni Education	67.8%	48.7%	1		8.00	<.01
Formal tx	26.4%	17.9%	1		1.72	.19
EOU (frequent)	6.9%	39.8%	1		15.75	<.001

Note: *N = *165; SDS = Severity of Dependence Scale; EOU = Exposure to other users.

### Logistic Regression

Hypothesis 1 predicted that unsuccessful quitting would be associated with several demographic variables, including younger age, male gender, being unmarried, and having less education. To test this hypothesis, demographic variables were entered into a binary logistic regression analysis. Although ethnicity was another demographic variable of potential interest, this was not included in the analysis due to the low number of individuals of non Anglo/Celtic background, as typically found in Australian studies of this type. Predictor variables included age, gender, marital status (those living with a partner also were categorized as being married), and education (at least some university education versus no university education). Results of the analysis are presented in Table 2. Hypothesis 1 was partially supported, with lower education being a significant predictor of unsuccessful quitting (OR = 0.34). No other demographic variable significantly predicted quitting success.

**Table 2:** Logistic Regressions Examining Demographic and Cannabis-Related Predictors of Quitting Status

Predictor	Odds ratio	**95% confidence interval**	
		Lower	Upper
Demographic			
Age	1.01	0.98	1.35
Gender	0.64	0.32	1.26
Marriage (yes/no)	0.74	0.34	1.60
University (yes/no)	0.34*	0.16	0.70
Cannabis-Related			
Age/initiation	1.07	0.97	1.19
Use (days/wk)	0.90	0.76	1.06
Frequent exposure (yes/no)	6.04***	2.21	16.55
Previous quit attempts	1.01	0.98	1.04
SDS	0.53*	0.32	0.88
Formal Treatment (yes/no)	0.38*	0.16	0.92

Demographics regression Nagelkerke *R*^2 ^= .10, *p *<.001. Cannabis-related variables regression Nagelkerke *R*^2 ^= .24, *p *<.001. Unsuccessful quitters coded as 0; successful quitters coded as 1.

Gender: males coded as 0, females coded as 1; Marriage: not married coded as 0, married coded

as 1; University: no coded as 0, yes coded as 1; Frequent exposure: no coded as 0, yes coded as 1.

SDS = Severity of Dependence Scale. Interpretation of binary logistic regression depends on

whether the predictor is categorical or continuous. The highest option is the reference point for categorical variables, whereas the lowest option is the reference point for continuous variables.

Thus, odds ratios below 1 indicate successful quitters scored lower than unsuccessful quitters on a variable when it is continuous, and that successful quitters scored higher on a variable when it is categorical. Odds ratios above 1 indicate that successful quitters scored higher than unsuccessful quitters on a variable when it is continuous, and that successful quitters scored lower on a variable when it is categorical. *p < .05.

Hypothesis 2 predicted that cannabis-related variables including higher use, higher dependence, and higher exposure to other cannabis users would be predictive of unsuccessful quitting. A second logistic regression analysis was conducted to test this hypothesis (see Table 2). Along with the variables hypothesized to predict quitting status, three other variables were entered into this analysis for exploratory purposes, including age of initiation, having previously sought formal treatment (yes/no), and number of previous quit attempts. In partial support of the hypothesis, higher exposure to other users and higher cannabis dependence prior to the most recent quit attempt significantly predicted unsuccessful quitting. Higher use prior to the most recent quit attempt did not significantly predict quitting status after controlling for other variables in the analysis. However, one additional variable, not having sought formal treatment, significantly predicted unsuccessful quitting.

### Principal Components Analysis

In order to reduce the probability of making Type 1 errors, and to identify themes among CBT and MI strategies, the 18 additional items targeting quitting strategies were entered into a principal components analysis, rather than being examined individually (see Table 3). A direct oblimin rotation was applied and variables with factor loadings higher than .50 were retained. Outlying variables and variables with cross-loadings exceeding .30 were removed from the analysis. This resulted in the removal of three variables ("I set goals for or relating to quitting"; "I made lifestyle changes"; and "I delayed using cannabis for a period of time when experiencing a craving"). A hybrid approach was used to determine the number of components to retain. Cattell's scree plot [33] suggested two components, while Kaiser's eigenvalues-greater-than-one rule [34] and a parallel analysis [35] both indicated four components should be retained. Based on this, and the high interpretability of the solution, four components were retained. The solution accounted for 64% of the variance in CBT and MI strategies. The derived components were named *Stimulus Removal *(Cronbach's α = .83), *Motivation Enhancement *(Cronbach's α = .84), *(lack of) Distraction *(Cronbach's α = .76), and *(lack of) Coping *(Cronbach's α = .72). Following the recommendations of Tabachnick and Fidell [36], pattern matrix loadings are reported. As can be seen from Table 3 components 3 and 4 only contained negative item loadings; thus higher scores on these components reflect lesser degrees of distraction and coping. Table 4 shows correlations among components as well as group means on component scores computed directly from the principal component analysis using the regression approach.

**Table 3:** Direct Oblimin (Δ = 0) Rotated Principal Component Loadings for CBT and MI Quitting Strategies Items

Variables	Component 1 Stimulus Removal	Component 2 Motivation Enhancement	Component 3 (lack of) Distraction	Component 4 (lack of) Coping
I avoided people who use cannabis	**.82**	-.24	.13	-.10
I avoided situations where people were likely to be using cannabis	**.79**	-.27	.12	-.16
I removed all the cannabis from my house	**.72**	.26	-.07	.13
I threw out my bong, rollies, or other smoking equipment	**.64**	.14	-.14	-.14
I avoided environments I associate with using cannabis	**.69**	-.07	.18	-.15
I discouraged anyone from using cannabis in my home	**.56**	.10	.02	.09
I thought about the benefits of quitting cannabis	-.02	**.81**	.10	-.11
I thought about the things I don't like about using cannabis	-.03	**.85**	.06	-.09
I thought about the future negative consequences of using cannabis	.18	**.79**	-.01	-.04
I did an activity to distract me	.10	.06	**-.84**	-.01
I did an activity that cannot physically be done at the same time as using cannabis	-.01	.04	**-.85**	-.03
I thought calming thoughts	-.03	.10	.15	**-.61**
I set up a plan for coping with situations in which I might be tempted to use cannabis	.13	-.13	.25	**-.73**
I found other ways to cope with stress and other negative emotions	.10	.01	.23	**-.77**
I found other ways to relax	.04	.11	.17	**-.70**

Following the recommendations of Tabachnick and Fidell [36], pattern matrix loadings are reported. These values are partial correlations between the scale items and the components, after controlling for the variance shared by the other retained components. Loadings > .30 appear in bold.

**Table 4:** Correlations Among Components in the Analysis and Group Means (SD) on Component (z) Scores

	Quitter Mean (*SD*)	Non-Quitter Mean (*SD*)	1	2	3	4
Stimulus Removal (1)	.09 (1.11)	-.10 (0.85)	-			
Motivation Enhancement (2)	-.15 (1.06)	.17 (0.91)	.18*	-		
(lack of) Distraction (3)	.07 (1.07)	-.08 (0.90)	-.19*	-.18*	-	
(lack of) Coping (4)	-.18 (1.08)	0.20 (0.86)	-.37**	-.21**	.30**	-

* p < .05, ** *p *< .01. Component scores are a weighted linear combination of *z *scores on the items of which the component is comprised.

### MANOVA

Hypothesis 3 predicted that successful quitters would use CBT and MI-related quitting strategies to a higher degree than would unsuccessful quitters. To test this hypothesis, successful and unsuccessful quitters were compared on the four CBT/MI components derived from the factor analysis, as well as the behavioral and experiential subscales of the Processes of Change measure, using MANOVA. The MANOVA was significant, *F *(6,158) = 3.30, *p *< .01; however, follow-up univariate tests only partially supported the hypothesis. While successful quitters scored significantly lower on (lack of) Coping, indicating they used significantly more coping strategies, they also scored significantly lower on Motivation Enhancement, indicating they used significantly fewer motivation enhancement strategies than did unsuccessful quitters. Other tests of univariate effects were non-significant (see Table 5).

**Table 5:** Univariate Tests for MANOVAs on Quitting Strategies and Distress

Dependent Variable	*df*	*df error*	*F*	*p*	η_p_^2^
Quitting Strategies (Wilks' λ = 0.90)	6	158	3.30	<.01	.11
POC Behavioral	1	163	2.41	.12	.00
POC Experiential	1	163	0.26	.61	.02
Stimulus Removal	1	163	1.41	.12	.01
Motivation Enhancement	1	163	4.47	.04	.03
(lack of) Distraction	1	163	1.02	.31	.01
(lack of) Coping	1	163	6.14	.01	.04
Distress (Wilks' λ = 0.93)	3	161	4.27	<.01	.07
Depression	1	163	10.25	<.01	.06
Anxiety	1	163	1.34	.25	.01
Stress	1	163	7.68	<.01	.05

Note: η_p_^2 ^= partial Eta squared; POC = Processes of Change.

The final hypothesis predicted that unsuccessful quitters would report significantly higher levels of depression, anxiety and stress. A second MANOVA was conducted to test this hypothesis. The MANOVA was significant, *F *(3, 161) = 4.27, p < .01, and univariate tests indicated partial support of the hypothesis, with unsuccessful quitters reporting significantly higher levels of depression and stress, but not anxiety, although the effect for anxiety was in the hypothesized direction. Table 5 provides details of the analysis.

## Discussion

The current study compared quitting strategies and personal characteristics of former regular cannabis users who had been abstinent for at least a year with current regular cannabis users who had made at least one unsuccessful attempt to quit. Based on previous research on other populations of cannabis users, it was hypothesized that the demographic characteristics of being younger, male, unmarried, and less educated would be associated with unsuccessful quitting. This hypothesis was partially supported with lower education being the only significant demographic predictor of unsuccessful quitting. The finding regarding education is consistent with previous research on cannabis use cessation [8,11] and likely relates to factors associated with the link between low education and substance use [37]. These may include having less developed problem solving/coping skills [38] and/or an impulsive decision making style [39], which may both encourage substance use and also make quitting more difficult.

Inconsistent with previous research [10,18], being unmarried, younger, and male were not significantly associated with unsuccessful quitting. However, around 30% of successful quitters were married compared with around 23% of non-quitters, suggesting a trend in the expected direction. The lack of effect for age may be due to the positive correlation between age and levels of cannabis use and dependence, which would likely counteract any relationship between older age and ability to quit. While the lack of effect for gender is inconsistent with previous research on cannabis use cessation, this is the first study to explicitly compare successful and unsuccessful cannabis quitters in a community setting. Studies focusing on tobacco quitting within a community setting have found some evidence that males are more likely to succeed at quit attempts than are females [40]. Therefore, individuals attempting to quit cannabis in a community setting may differ from treatment samples. Future studies could further explore this possibility.

Hypothesis 2 predicted that cannabis-related variables including higher use, higher dependence, and higher exposure to other cannabis users would be predictive of unsuccessful quitting. Consistent with previous research, both higher dependence and higher exposure to other cannabis users were associated with unsuccessful quitting [9,12,13]. Overall, having frequent exposure to other users was the strongest predictor of unsuccessful quitting (OR = 6.04). Inconsistent with previous research and study hypotheses, frequency of cannabis use did not predict quitting status. Unsuccessful quitters, however, did report somewhat higher frequency of use prior to their most recent quit attempt (4.81 days per week compared with 3.79 days for successful quitters). Inter-correlations with other variables included in the analysis may account for the non-significant finding. Unsuccessful quitters were significantly less likely to have had professional treatment for cannabis use in the past. Therefore, having professional treatment may contribute to successful quitting.

Hypothesis 3 predicted that successful quitters would use CBT and MI-related quitting strategies to a higher degree than would unsuccessful quitters. Behavioral and experiential strategies in general, measured using the Processes of Change questionnaire, did not distinguish between successful and unsuccessful quitters. However, using principal components analysis on the 18 additional strategies, four types of cannabis quitting strategies were found among this sample of 165 cannabis (ex)users: Stimulus Removal, Motivation Enhancement, (lack of) Distraction, and (lack of) Coping. Unsuccessful quitters were significantly more likely to use motivation enhancement strategies in their attempt to quit, while successful quitters were significantly more likely to use coping strategies to quit. Successful and unsuccessful quitters used stimulus removal and distraction techniques at equal rates. Contrary to study hypotheses, not all motivational and cognitive-behavioral strategies were found to predict successful quitting. In fact, only frequent use of coping strategies predicted successful quitting. Individuals who thought calming thoughts, found other ways to relax, found other ways to cope with aversive emotions, and set up a plan to use these techniques were more likely to maintain abstinence.

Unexpectedly, frequent engagement in motivational enhancement techniques was associated with a greater chance of failing to quit. This finding is at odds with research that has found brief motivational interventions can produce positive outcomes among treatment seekers [5]. Examination of the component scores suggests that individuals who relied upon motivational strategies may have done so to the detriment of using stimulus removal and coping techniques. Unsuccessful quitters had stimulus removal scores below the mean, and (lack of) coping scores above the mean. Thus, it may not be that using motivational strategies is problematic *per se*, but more that using them as the primary method for quitting puts individuals at a greater risk of quitting failure. Interestingly, Litt and colleagues [5] found that combined MI and CBT and MI alone produced equivalent increases in utilization of cognitive behavioral quitting strategies. Thus, the effective ingredient for reducing cannabis use in motivational enhancement may be skill utilization, rather than utilization of motivational techniques. Theoretically, one would only expect motivational techniques to be necessary during pre-action stages and that during action stages individuals may need to draw from a larger repertoire of quitting strategies. In order to confirm this theory, future research needs to be concerned with monitoring strategies used during various stages of change and evaluating how effective they are during those particular stages. To date, few studies have reported on the major treatment targets or mechanisms of effectiveness of motivational interviewing or cognitive-behavioral therapy for engendering enduring behavior change.

Stimulus removal and distraction were not found to differentiate successful from unsuccessful quitters. The former finding may be due to how often the current sample engaged in the strategy. Approximately 70% of the sample used stimulus removal occasionally or more often during their most recent quit attempt. Given that most people engaged in stimulus removal, it may be that the strategy is necessary for successful quitting, but not sufficient. Boyd and colleagues [14] found that the four most frequently cited quitting strategies among a sample of non-treatment seeking cannabis smokers involved stimulus removal. Likewise, these variables were not related to the duration of participants' longest quit attempt. Overall, the findings on quitting strategies are consistent with previous research suggesting that higher use of CBT-related strategies predicts better outcomes for reducing cannabis use [5]. The findings extend previous research by distinguishing between different types of strategies, and highlighting the potential importance of those that relate to coping.

Hypothesis 4 predicted that unsuccessful quitters would report significantly higher levels of depression, anxiety and stress. This hypothesis was partially supported, with unsuccessful quitters reporting significantly higher levels of depression and stress. This outcome is generally consistent with previous research, which has found that psychological distress is predictive of relapse among treatment samples, and continued use among community members [9,19-22]. Interestingly, while successful quitters' depression and stress scores were significantly lower than unsuccessful quitters' scores, they continued to be quite high. Successful quitters reported severe levels of depression and stress, while unsuccessful quitters reported extremely severe levels of depression and stress. Furthermore, both successful and unsuccessful quitters reported extremely severe levels of anxiety at the time of survey completion. Thus, both groups experienced levels of distress that likely impacted upon their daily functioning and such high amounts of distress may trigger future use among successful quitters. These findings reinforce the potential importance of taking comorbid psychological problems into account when developing treatments designed to assist individuals to quit cannabis, and also highlight the need for long-term follow-up of quitters to see how long quitting endures in the context of severe distress.

The findings of the current study have implications for future research and interventions for cannabis use. While this was a relatively well educated sample, with almost half of the unsuccessful quitters having at least some tertiary education, future studies could further explore the association between education level and relapse by establishing whether the link is mediated by factors such as higher impulsivity and lower problem solving skills, and by determining which interventions are most effective and appealing to individuals with lower levels of education. Reducing exposure to other users and developing methods of coping with such exposures without using cannabis may also contribute to more successful treatment. Coping techniques were the most important strategies contributing to successful quitting, suggesting that individuals who fail to quit may do so because they have trouble coping with distress. Treatments for cannabis use may, therefore, increase their effectiveness through placing greater emphasis on coping skills, and also addressing the source of distress. Relating to this, given that higher levels of stress and depression were associated with unsuccessful quitting, interventions addressing comorbid psychological health problems should be developed and tested for effectiveness in future research.

The current study is limited by its retrospective design. Future studies examining the characteristics of successful and unsuccessful quitters may benefit from collecting data prospectively. A further consideration is that data relating to cannabis use and quitting were collected using a self-report method; validation of abstinence from cannabis through methods such as urinalysis would be a desirable addition to a study of this kind. Sample size and the possibility of Type 2 error should be considered with regard to analyses where trends toward significant differences between successful and unsuccessful quitters were apparent. Finally, although days per week of cannabis use was not observed to be a significant predictor of quitting status in the logistic regression analysis once other variables were held constant, this variable did significantly predict quitting status when examined individually. The opposite pattern was observed for the variable assessing whether formal treatment for cannabis use was sought (i.e., this variable did not significantly predict quitting status when examined individually, but was a significant predictor in the logistic regression analysis). Results relating to these variables should thus be interpreted with consideration that suppressor effects may have been present.

## Conclusion

This study found that lower education, higher cannabis dependence, higher exposure to other cannabis users, lower use of coping-related strategies, and psychological distress were associated with unsuccessful attempts to quit cannabis among a community sample. Future interventions for cannabis use may show increased success through tailoring treatments to target these demographics, and through placing an emphasis on the development of coping skills.

## Competing interests

The authors declare that they have no competing interests.

## Authors' contributions

SR contributed to the research design, data collection and analysis, and writing of the manuscript. MN contributed to the research design, data analysis and writing of the manuscript. JC contributed to the research design and writing of the manuscript. All authors read and approved the final manuscript.

## Supplementary Material

Additional file 1 **Correlation matrix**. Correlations among continuous variables in the analyses. Click here for file
