# Supplementary material for: Successful and unsuccessful cannabis quitters: Comparing group characteristics and quitting strategies
Source: Subst Abuse Treat Prev Policy. 2011 Nov 11;6:30. doi: 10.1186/1747-597X-6-30 (PMC3229433; doi:10.1186/1747-597X-6-30)
Supplement: Additional file 1 — Correlation matrix. Correlations among continuous variables in the analyses. [file 1747-597X-6-30-S1.DOCX]

*Correlations among Continuous Variables in the Analyses*

| Measure | 1 | 2 | 3 | 4 | 5 | 6 | 7 8 9 10 |
| --- | --- | --- | --- | --- | --- | --- | --- |
| Age (1) | - |  |  |  |  |  |  |
| Age/initiation (2) | .34** | - |  |  |  |  |  |
| Use (days/wk) (3) | .32** | -.25** | - |  |  |  |  |
| Quit attempts (4) | .14 | -.08 | .20* | - |  |  |  |
| SDS (5) | .29** | .09 | .44** | .27** | - |  |  |
| POC Behav. (6) | .07 | .22** | -.03 | .12 | .45** | - |  |
| POC Exper. (7) | .09 | .22** | -.03 | .11 | .48** | .75** | - |
| Depression (8) | -.10 | .11 | .04 | .08 | .33** | .30** | .32** - |
| Anxiety (9) | -.18* | .12 | -.08 | .04 | .21** | .29** | .35** .68** - |
| Stress (10) | -.04 | .10 | .05 | .09 | .40** | .35** | .36** .80** .78** - |

**p* <.05 ***p*< .01 ****p*<.001; EOU = Exposure to other users; SDS = Severity of Dependence Scale; POC

Behav. = Processes of Change Behavioral; POC Exper.. = Processes of Change Experiential.
